# Measuring and Modeling the Effect of Audio on Human Focus in Everyday Environments Using Brain-Computer Interface Technology

**DOI:** 10.3389/fncom.2021.760561

**Published:** 2022-01-27

**Authors:** Aia Haruvi, Ronen Kopito, Noa Brande-Eilat, Shai Kalev, Eitan Kay, Daniel Furman

**Affiliations:** Arctop Inc., San Francisco, CA, United States

**Keywords:** artificial intelligence, audio, brain-computer interface, focus, human

## Abstract

The goal of this study was to investigate the effect of audio listened to through headphones on subjectively reported human focus levels, and to identify through objective measures the properties that contribute most to increasing and decreasing focus in people within their regular, everyday environment. Participants (*N* = 62, 18–65 years) performed various tasks on a tablet computer while listening to either no audio (silence), popular audio playlists designed to increase focus (pre-recorded music arranged in a particular sequence of songs), or engineered soundscapes that were personalized to individual listeners (digital audio composed in real-time based on input parameters such as heart rate, time of day, location, etc.). Audio stimuli were delivered to participants through headphones while their brain signals were simultaneously recorded by a portable electroencephalography headband. Participants completed four 1-h long sessions at home during which different audio played continuously in the background. Using brain-computer interface technology for brain decoding and based on an individual’s self-report of their focus, we obtained individual focus levels over time and used this data to analyze the effects of various properties of the sounds contained in the audio content. We found that while participants were working, personalized soundscapes increased their focus significantly above silence (*p* = 0.008), while music playlists did not have a significant effect. For the young adult demographic (18–36 years), all audio tested was significantly better than silence at producing focus (*p* = 0.001–0.009). Personalized soundscapes increased focus the most relative to silence, but playlists of pre-recorded songs also increased focus significantly during specific time intervals. Ultimately we found it is possible to accurately predict human focus levels *a priori* based on physical properties of audio content. We then applied this finding to compare between music genres and revealed that classical music, engineered soundscapes, and natural sounds were the best genres for increasing focus, while pop and hip-hop were the worst. These insights can enable human and artificial intelligence composers to produce increases or decreases in listener focus with high temporal (millisecond) precision. Future research will include real-time adaptation of audio for other functional objectives beyond affecting focus, such as affecting listener enjoyment, drowsiness, stress and memory.

## Introduction

### The Effect of Sound on Human Experience

Sounds are all around us, from natural sounds like the wind, to engineered sounds like music. It is well-established that sounds have a major influence on the human brain and consequently, human experience (Levitin, 2006; Sacks, 2010). Sounds can reduce stress (Davis and Thaut, 1989), support learning and memory formation (Hallam et al., 2002), improve mood (Chanda and Levitin, 2013), and increase motivation (Salimpoor et al., 2015). Sounds can also do the opposite and create aversive experiences (Schreiber and Kahneman, 2000; Zald and Pardo, 2002; Kumar et al., 2012). One of the most significant effects of sounds is to impact focus. Focus is commonly demanded by tasks of daily living and work, and in these areas sounds experienced as audio through headphones, earbuds or speakers offer a safe way to increase focus levels and productivity. However, sounds can be both beneficial or distracting and previous results have been inconclusive in determining the reasons why (de la Mora Velasco and Hirumi, 2020).

For example, it has been found that listening to music with lyrics while reading or working can decrease concentration or cognitive performance (Shih et al., 2012; Liu et al., 2021), while several studies have shown oppositely that natural-occurring sounds such as white noise, or highly composed sound such as classical music, can be beneficial for increasing focus and can even improve learning outcomes (Davies, 2000; Chou, 2010; Angwin et al., 2017; Gao et al., 2020). Therefore, one interesting question that emerges is: what are the specific properties of an audio experience that affect human focus levels the most? Additionally, studies have shown that the effect of audio is often subjective, where whether one likes a given sound or not is a key factor in its effect on their experience (Cassidy and Macdonald, 2009; Huang and Shih, 2011; Mori et al., 2014). Although this finding about the subjectivity of experience of audio reappears across many studies, psychophysical thresholds are known to exist and there are clearly natural laws governing much of the way humans hear and experience sound (Levitin et al., 2012; Nia et al., 2015; Washburne, 2020).

The potential of audio alone to increase focus, and the consumer demand for non-pharmaceutical tools that enable individuals to enhance their own ability to focus has recently led several companies (including Focus@Will, Endel, Brain.fm, Mubert, Enophone, Melodia, AIVA, and others) to develop audio content that is dedicated to increasing focus “on-demand.” These new audio forms include elements of white noise, music, and other sonic properties that are functionally combined to increase a listener’s focus and maintain high levels of focus over a long duration of time. One of the challenges in this field is to figure out the physical properties of sound that contribute to human experience the most so that design principles can be defined correctly to create audio that reliably achieves the goal of increasing focus, opposed to the inverse of causing distractions and impairing an individual’s ability to focus. Insights about audio properties therefore have been sought by commercial groups alongside academic groups in order to learn how to optimally enhance human focus.

Many scientific studies have explored this question and looked for the relationship between sound, music, and human experience using objective measures that empirically assess properties of audio and their emotional correlates. For example, Cheung et al. (2019) found that pleasure from music depends on states of expectation, such as a skipped rhythmic beat, which can either be pleasurable or discomforting depending on the listener’s specific circumstance. *Sweet Anticipation* (Huron, 2006) similarly maps how music evokes emotions within a theory of expectation and describes psychological mechanisms that are responsible for many people’s mixed responses to audio of various types. Other studies used machine learning methods to map from features of audio signals to emotions (Yang et al., 2008; Vempala and Russo, 2012; Brotzer et al., 2019; Cunningham et al., 2020; Hizlisoy et al., 2021). These machine learning studies to date have, however, only aimed to predict emotions based on the limited valence-arousal circumplex model, and as far as we know, no attempts have been made to predict human focus levels exclusively based on audio signal analysis.

One persistent obstacle to the field’s understanding has been studies that rely on data with a low temporal resolution. Since audio content and emotions can change fast, on the order of tens of milliseconds, the current lack of modeling tools capable of capturing quick, transient changes in human experience that accompany changes in sound is a major hindrance to progress (Larsen and Diener, 1992; Cowen and Keltner, 2017). Commonly, for example, reports are based on data where there is a single emotional label per song, while the song lasts 2–3 min and throughout it there are emotional dynamics that change dramatically. This mismatch of data can lead to conclusions being drawn from inadequately small amounts of samples, and worse than that, inaccurate emotional labels.

### Attention and Emotion Decoding From Brain Signal

Brain decoding technology offers an exceptional opportunity to solve this issue, since it enables an estimation for the experience dynamics at the same time resolution as focus phenomena occur. Using electroencephalogram (EEG) sensor data, which contains electrical brain activity measured from the scalp (non-invasive) on the order of hundreds of measurements per second, many studies have established that it is possible to capture fast changes in human emotions and experience, such as stress (Perez-Valero et al., 2021), arousal (Faller et al., 2019), fatigue (Hu and Yang, 2017), and happiness (Lin et al., 2017). Several studies have similarly shown the ability to capture focus and attentional state changes, affirming that this information is present in EEG sensor data (Jung et al., 1997; Hamadicharef et al., 2009; Micoulaud-Franchi et al., 2014; Tuckute et al., 2021). While brain decoding technology has been applied widely to study the effects of different types of stimuli (e.g., visual, tactile, and auditory) on human experience within a laboratory environment (Bhatti et al., 2016; Shahabi and Moghimi, 2016; Asif et al., 2019), as far as we know, it has not been applied to study the joint effects of audio and focus at the high temporal resolution needed to explain both phenomena as they occur in people’s natural, everyday environments.

In recent years, progress in the development of consumer brain-computer interface wearable technology such as non-clinical, non-invasive EEG sensors (such as Muse, NeuroSky, Emotiv, Bitbrain, etc.), which are intended for personal use, has led to new research paradigms. Now it is possible to use comfortable, affordable, wireless, and easy-to-use at-home measurement devices to collect neuroscientific data “in-the-wild” at a large scale, which opens up for the first time the opportunity to measure brain responses from diverse audiences within their natural habitats. Many of the wearable brain-computer interface devices offer real-time decoding outputs that are derived from the raw electrophysiological sensor data. These “off-the-shelf” decoding outputs include attention, relaxation, and other states (Rebolledo-Mendez et al., 2009; Liu et al., 2013; González et al., 2015; Abiri et al., 2019; Bird et al., 2019). It is important to note, however, that although decoder algorithms have existed in the market for consumer uses for several years, verifying their reliability to accurately capture attention, valence, arousal, stress, and other attributes of human experience at a high temporal resolution, advanced research quality has remained a challenge.

### Combining Brain–Computer Interface Technology With Audio Tests to Decode Focus

In the current study, we used a brain-computer interface algorithm package, Neuos™ Software Development Kit (Neuos SDK from Arctop Inc.), for processing data from portable fabric EEG headbands (Muse-S from Interaxon Inc.) in order to measure human focus levels in individuals performing tasks at home while they listened to different audio content through headphones. The ground truth focus levels we used were based on each individual’s subjective, self-report. Since the Neuos SDK product is a relatively new technology, we first evaluated the validity of the focus outputs within the experimental conditions. Then, once the algorithm outputs were found to be reliable and accurate in this context, we use the focus data to compare effects of different sound stimuli on individuals as they carried out different tasks.

Next, we exploited the high temporal resolution of the decoded data to map between raw audio signals and the focus dynamics. Based on this mapping, we built a model that takes as an input an audio file and predicts from the properties of sound in the audio the corresponding focus levels that human listeners will experience. This high resolution modeling enables us to compare between new songs, various sounds, and between genres to gain additional insights about the nature of audio stimuli that drive human focus the most. These insights can help produce optimal playlists to increase focus for general audiences, improve design of custom soundscapes for work and learning environments, and even adapt audio in real-time based on an individual’s focus levels to allow them precise influence over their own mental state.

## Materials and Methods

### Participants

Sixty-two participants (40 males, 22 females, 18−65 years), completed four sessions over a single week at their own home. All participants were recruited from an opt-in screening panel and were distributed approximately evenly across the five major regions of the continental United States (Northeast, Southwest, West, Southeast, and Midwest). Only participants who reported normal hearing, normal vision, or vision that was corrected to normal with contact lenses, were included. We excluded volunteers who reported using medication that might influence the experiment and who reported neurological or psychiatric conditions that could influence results. Participants were native English speakers and a written informed consent was obtained from each participant prior to their participation. Participants received compensation for their time.

### Paradigm

#### Tasks

**FIGURE 1:**
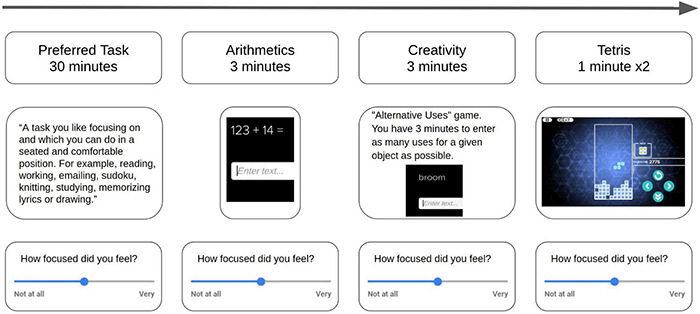
Schematic illustration of the paradigm in each recording session. Each session started with 30 minutes of a task selected by the participant (“Preferred Task”), followed by 3 min of arithmetics exercises, 3 min of a creativity task, and two levels of Tetris the video game (each level lasted 1 min regardless of performance). After each task, participants answered a survey where they reported on aspects of their experience (e.g., focus, enjoyment, and stress) using linear scale sliders from “Not at all” (0) to “Very” (1). The short tasks were used as calibration tasks and the “Preferred Task” was the test task.

Participants performed various tasks within a mobile Android app (“Neuos Central” by Arctop Inc.) while listening to one of three types of audio and wearing a brain signal measuring headband (four-channel EEG Muse-S device by Interaxon Inc.). Each participant received a kit at their home by mail that included all the equipment needed to participate, including over-ear (Sony Group Corporation) headphones, headband (Interaxon Inc.) and tablet computer (Samsung Electronics Co., Ltd.) with the mobile app installed. Participants recorded four 1 h long sessions, while listening to different audio types. Sessions included 30 min of a “Preferred Task” – a task chosen by the participant – followed by short tasks (“calibration tasks”). These short tasks included video games (Tetris), math problems (Arithmetics), and word problems (Creativity) that were used to calibrate the sensors to the individual. Participants were assigned to groups according to a pseudorandom schedule that controlled for potential sequence effects of the tasks and different audio types (Figure 1). The short tasks calibrated the Neuos SDK decoding algorithms to a validated performance level for each participant and afterwards the individually validated model was used to measure each participant’s focus level across the Preferred Task.

Participants were instructed to choose a Preferred Task that they could perform in a seated position while listening to audio through the headphones, and which they would be happy to repeat in all four sessions. For example, Preferred Tasks that were chosen included working, reading, knitting and solving Sudoku puzzles. At the end of each task the participants self-reported their experience through a survey in the app which used linearly scaled slider buttons to quantify experience along several dimensions (e.g., focus level, enjoyment, stress, motivation, etc.). For the Preferred Task, the survey included reporting on their focus level during the first and second half of the task separately, resulting in six self-reported quantitative focus labels per session (Preferred Task: two labels, arithmetics: one label, creativity: one label, tetris: two labels).

#### Audio Stimuli

Each participant experienced four audio conditions over the 4 days of the study: two music playlists by leading digital service providers Spotify and Apple (downloaded September 2020), one personalized soundscape engineered by Endel, and silence (no audible sounds). We selected Spotify’s “Focus Flow” playlist and Apple Music’s “Pure Focus” playlist to represent the category of pre-recorded audio designed to increase listener focus. For soundscapes we selected the mobile app Endel to represent the category of real-time, engineered audio that contains a mixture of noise and musical properties. The Endel app “Focus” soundscape was used by each participant on their own mobile device. All audio conditions were instrumental and did not include singing or any audible lyrics. For the condition of silence, participants wore headphones exactly as they did in the audio conditions but no music or audible sounds of any kind were played and no soundscape was generated – participants simply completed the session in a quiet environment.

### Data Processing

#### Data Acquisition

**FIGURE 2:**
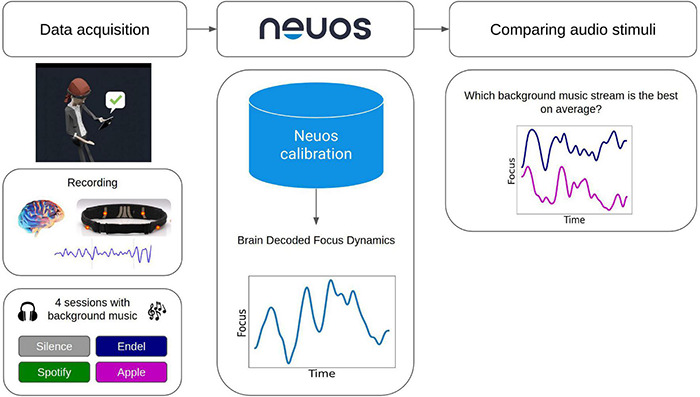
Schematic illustration of the data processing pipeline. Data acquisition included at-home recordings of four sessions, each with a different background sound type. Arctop’s Neuos SDK brain decoding technology package was used to predict the focus dynamics at a rate of 5 Hz. Obtaining the brain decoded focus dynamics synchronously with the sound content enables comparison of focus levels correlated with different physical properties of sound.

While participants were listening to audio stimuli and engaging in the experimental tasks, their electrical brain activity was recorded using a fabric electroencephalograph (EEG) headband that was wireless and weighed 41 g (Muse-S device by Interaxon Inc.). The headband included four dry EEG sensors (sampling rate: 256 Hz), photoplethysmography (PPG) sensors (for heart rate) and motion sensors (gyroscope, accelerometer). The brain-measuring EEG sensors were located on the scalp at two frontal channels (AF7 and AF8) and two temporal channels (TP9 and TP10), with the reference channel at Fpz. The headbands were put on by participants themselves with the assistance of a quality control screen in the app that started each session by giving participants real-time feedback on the signal quality of their headband and made it easy for them to adjust the headband appropriately to acquire the optimal signal (Figure 2). No technicians or other support staff assisted in the placement of the headbands – the process was completely automated by the in-app prompts within the “Neuos Central” app, freeing the participants to complete sessions at any time or place of their choosing.

#### Brain Based Models of Focus

Brain decoding algorithms designed for real-time brain-computer interfacing (Neuos SDK) was used to transform raw sensor data into predicted focus dynamics with a time resolution of 5 Hz (Figure 2). The short tasks (games, word and math problems) were used for calibration of the sensors to the focus dynamics of each individual, and then a calibrated model per participant was applied on the Preferred Task data across all days. The full analysis procedure included data exclusion, preprocessing, feature extraction, and applying machine learning models to transform raw data features to decoded focus dynamics is explained below.

##### Data Exclusion

Since brain activity and survey data was collected in participants’ own homes, as a quality control step before preprocessing we first validated the data with respect to headband positioning (to confirm it was correctly placed), survey responses (to make sure participants followed the instructions properly) and internet issues (which occasionally resulted in missing measurements). This procedure led to 11 participants data being excluded from further analysis due to the following reasons:

1. Three participants were excluded due to misplacement of the headband which caused excessive noise in their recorded data. To identify the misplacement we simply extracted the standard deviation of the raw signal (SD > 500 reflects a misplaced channel). Supplementary Figure 1 shows three examples of the raw data of problematic participants vs. three examples of valid participants.
2. Two participants were excluded for not following the instructions correctly during the Preferred Task.
3. Six participants were excluded due to persistent internet issues which caused missing or disrupted data.

After data exclusion, a total of 51 participants (mean age = 36, SD = 8, 17 females and 34 males) were included in the experimental analysis.

##### Preprocessing

A band-pass filter (0.5–70 Hz) was applied to each channel together with a notch filter (60 Hz) to remove line noise. During the performed tasks, 5 s of EEG data segments were extracted from the filtered signal using a sliding window with a stride of 200 ms (96% overlap) to obtain a time resolution of 5 Hz of accurate focus measurements reliably across each task. Headband motion sensor (gyroscope) data were used to detect the motion state of each segment (static, medium or high movement) and segments with substantial movements (medium or high) during the short calibration tasks were automatically excluded. During the Preferred Tasks, all segments were included regardless of movement state in order to obtain continuous dynamics for the full 30 min.

To validate that the differences in responses to each audio stream were not due to differences in movement patterns evoked by the audio, we compared the motion statistics between audio types. Supplementary Figure 2C shows that during the Preferred Task, participants were stationary 91% of the time and similarly for all audio types (Supplementary Figure 2D). To address eye blinks, which are a normal human function that can corrupt EEG data, we calculated the number of blinks in each EEG segment. Supplementary Figure 2A shows the histogram of the blink rate across participants (average blinks per minute = 16±6). Supplementary Figure 2B shows similar rates of eye blinks for all audio streams, ruling out the possibility that the differences in the effects of audio were due to differences in eye blink patterns which may have introduced artifacts to the decoded data.

##### Feature Extraction

From each EEG segment (epoch) a total of 124 features were extracted, then to handle outliers and avoid extreme values each feature underwent a programmatic trimming procedure that denoised high and low values (extreme values were defined as above or below 2 SD from the mean). The following features were used:

a. Average power spectrum features – each segment was transformed to the frequency domain using Welch method, and for each channel, the average power in different frequency bands was calculated (0.5–4, 4–8, 8–12.5, 12.5–30, 30–47, 52–70, and 30–70 Hz) – a total of 4 channels × 7 bands = 28 features.
b. Ratios between average power for spatial symmetric channels (frontal: $\frac{AF7}{AF8}$ and temporal:$\frac{TP9}{TP10}$) – a total of 2 pairs × 7 bands = 14 features.
c. Power spectrum interactions – the power spectrum ratio between bands ($\frac{alpha}{delta}$,$\frac{beta}{theta}$,$\frac{theta}{alpha}$ (Barachant, 2017) and engagement index ($\frac{beta}{alpha+theta}$) (Pope et al., 1995) – a total of 4 channels × 4 interactions = 16 features.
d. Pairwise Pearson correlations between channels in the above frequency bands – 6 pairs × 7 bands = 42 features.
e. Time domain features – for each channel, the first four moments (average, standard deviation, skewness, and kurtosis), entropy and number of zero-crossing points – total of 4 channels × 6 types = 24 features.

##### Machine Learning Models

Average features were calculated across each short task (games, word and math problems) for all valid participants and from all days, resulting in 816 focus-ranked tasks (51 participants × 4 sessions × 4 ranked subtasks per session). Then, in a cross validation procedure, multiple random forest regression models provided by the Neuos SDK software package were trained on random subsets of participants (80%) to predict the self-reported focus based on the computed features. For each participant, from the subset of models for which their data were not part of training, the single best model was selected based on the Pearson correlation between the model prediction and the self-reported focus by that participant during the short calibration tasks. The selected regression model was then applied to EEG segment data during their Preferred Task 30 min recordings to get a continuous brain-decoded gradient of focus dynamics that was accurate.

**FIGURE 3:**
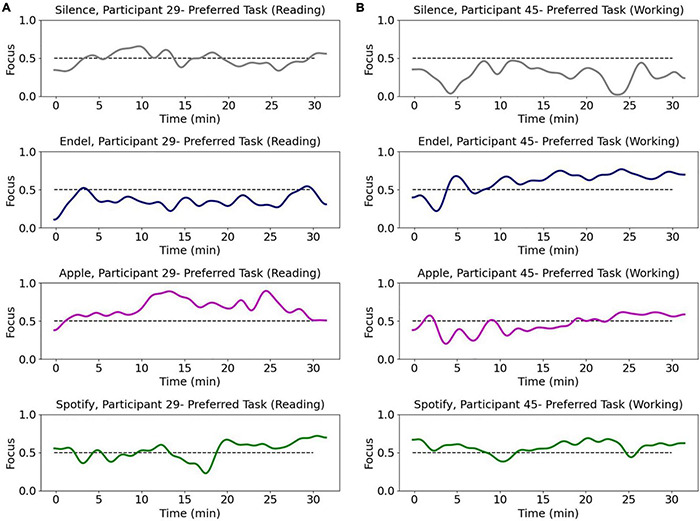
Brain data based focus model dynamics of two representative participants during the Preferred Task performed at each of the four sessions. Each row represents a session with a different sound stream playing in the background as participants perform their chosen task. Each session included 30 min (*x*-axis = time in minutes) of a “Preferred Task” over which their focus level (*y*-axis = decoded focus) was measured. Participant 29 **(A)** was reading while Participant 45 **(B)** was working.

A Gaussian filter was used to smooth the dynamics of the brain-decoded focus gradient and all of the presented results and statistical analysis in this paper are projections of the Gaussian filtered model outputs on the Preferred Task which was not part of the training and selection process for each participant. Figure 3 shows the resulting brain decoded focus levels of two representative participants across all four sessions during the Preferred Task period. Model performance was evaluated using Pearson correlation coefficient between the self-reported focus and the brain decoded focus values after thresholding the values, with the area under the ROC curve for binary classification of low/high focus (Figure 5).

Electroencephalogram signals are non-stationary and can change dramatically over time (Haartsen et al., 2020; Padilla-Buritica et al., 2020; Yang et al., 2021). To validate that the obtained focus dynamics were not influenced by the non-stationarity of the EEG signal or other forms of signal drift that can occur with electrophysiological measures, we compared the averaged focus levels across all sessions during the first 15 min of the Preferred Task to the last 15 min (Supplementary Figure 3). We found there was no significant difference between the segments and concluded that the signal processing methods were robust to this form of signal artifact.

#### Statistical Methods

For comparisons between average focus levels in response to the different audio streams, we calculated for each participant (*N* = 51) the median focus level while performing the Preferred Task and conducted a one-way repeated measures ANOVA (analysis of variance) test. Then, if *p* < 0.05, paired *t*-tests were applied *post hoc* to compare between pairs of audio streams using the Holm–Bonferroni correction. Time series statistical tests were applied to compare focus level dynamics and discover specific time periods where there was significant difference. A paired *t*-test was applied to each second between focus levels of two audio streams and the *p*-values were then corrected for multiple comparisons by setting a threshold for a minimum significant sequential time-samples. The threshold was determined by random permutations (1000 iterations) of participants’ conditions and repeating the statistical test, resulting in a distribution of significant sequential time samples. The threshold was set as the 95% percentile of the resultant distribution (Broday-Dvir et al., 2018).

#### Audio Signal Decomposition and Feature Extraction

**FIGURE 4:**
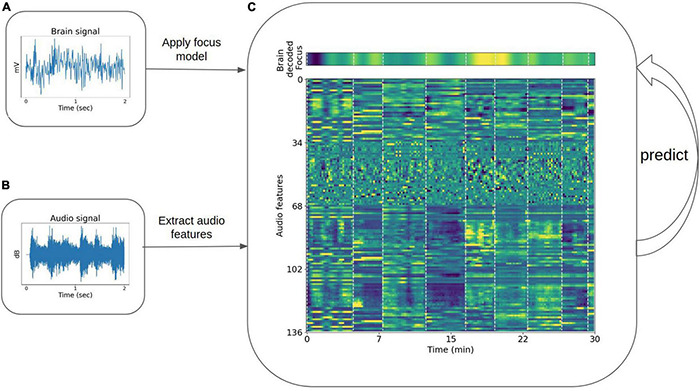
Diagram demonstrating the framework for correlation of time-series focus values with sound properties. **(A)** Example of a recorded brain data in microvolts (single channel of EEG) segment, which after applying the preprocessing and trained models on 30 min of recordings, transforms to the brain decoded focus dynamics [top **(C)]**. **(B)** Examples of a sound segment in decibels taken from one of the songs. [Bottom **(C)]** The sound features (*y*-axis) dynamics during 30 min of recordings (*x*-axis).

The pre-recorded music playlist conditions (Apple and Spotify) provided raw audio data that we used to obtain sound property dynamics in the time and frequency domain. These dynamics could then be correlated with the obtained focus dynamics as averaged across participants. Soundscape audio content and silence conditions were not used in this analysis because the soundscapes were produced in real-time personally for each participant, which limited the ability to apply sound property analysis appropriately across the data set, and the silent condition yielded no sound features (no microphones were used during the session from which miscellaneous sounds might have otherwise been extracted). The audio features were calculated for each playlist using Python’s library pyAudioAnalysis (Giannakopoulos, 2015), for example, the sound signal energy, spectral entropy, and chroma coefficients were extracted. The features were calculated in short-time windows of 50 ms with a sliding window of 25 ms. Basic statistics were then calculated over the sound features in windows of 30 s (e.g., mean and SD), resulting in 136 sound properties (link to full list). To enable mapping of audio features to the brain model, the brain decoded focus levels were averaged across participants and averaged in corresponding 30 s windows (Figure 4) to obtain a singular collective dynamic that could be used to predict focus from audio content with the same number of samples as the audio feature properties.

#### Obtaining the Sound Decoded Focus Model

To map the relationship between properties of the audio heard and focus levels measured directly from the brain, we applied principal component analysis (PCA) to reduce the dimensionality of the audio features (using 33 dimensions ultimately, which explained 95% of the data variance). We then trained a linear regression model to map between the transformed audio features and the averaged brain decoded focus levels. The training was done using backward features elimination where in each iteration the component with the smallest weight on average was eliminated. To evaluate model performance, training was done with a stratified cross validation procedure in which we divided the data set to training and validation according to the songs played (to avoid time dependency issues between the sound features). A total of 18 different songs were played during the pre-recorded playlists (8 songs for Apple, 10 songs for Spotify). In each iteration, 14 songs were used in training and 4 as validation (77/23%). For each sample and song, the audio decoded score is the average model predictions calculated across the models it was part of in the validation set.

## Results

### Brain-Measured Focus Levels Accurately Reflect Self-Reported Focus Levels

**FIGURE 5:**
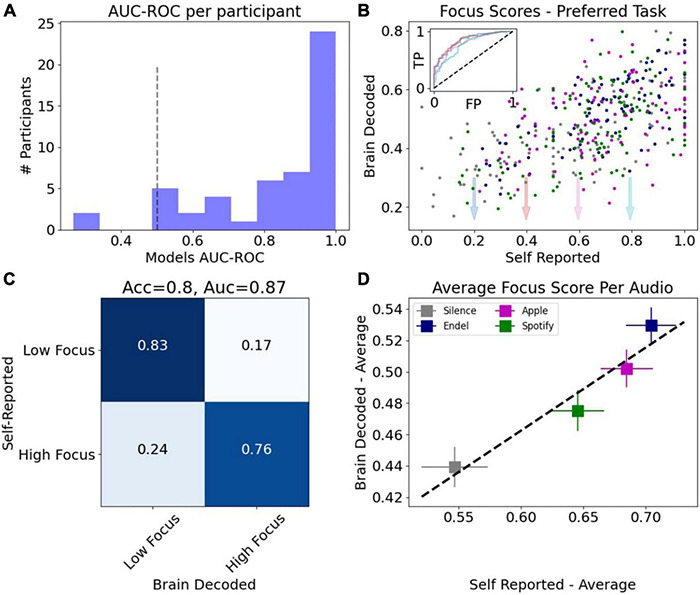
Validation of focus measurements derived from brain data. **(A)** Histogram of focus models performance on the test task per participant (*N* = 51), evaluated using the area under the ROC curve (AUC-ROC). Black dashed line marks chance level (0.5). **(B)** Average focus levels per preferred task events vs. self-reported focus resulted in Pearson correlation of 0.6. Inset shows ROC curves for different values of self-report threshold. **(C)** Confusion matrix after thresholding the focus score predictions and self-report. Classification scores for two-classes (low focus vs. high focus) are AUC = 0.87 (area under ROC curve), accuracy = 0.8. **(D)** Average brain decoded focus levels vs. average self-reported focus across the four sound types.

After calibration tasks established an initial model for each participant, and before comparing focus levels elicited by the different audio types, we validated that the underlying brain decoding technology was accurate and correctly calibrated by comparing between the brain-based focus predictions and the self-reported focus values during the test task (the “Preferred Task”). Figure 5A shows a histogram of the model performance per participant. The model is evaluated based on the AUC score (of the ROC curve) for prediction of self-reported focus during the Preferred Task (low-high focus) where the chance guessing level is 0.5 (black dashed line). The average result across participants obtained was ≪*cps*:*it* > *auc* < /*cps*:*it*≫ = 0.83 (*N* = 51, SD = 0.19), a strong validation of the brain-measured focus accuracy.

When aggregating tasks from all participants, the Pearson correlation between the brain decoded focus model and the self-reported focus was Corr(414) = 0.6, *p* < 10^–4^ (Figure 5B). The inset in Figure 5B shows the ROC curves for different values of self-reported threshold and the confusion matrix for one of these thresholds (0.4) resulted in an accuracy score of 0.8 (Figure 5C). Figure 5D shows the average brain decoded focus level for each audio type vs. the average self-reported score.

### Soundscapes Induce Higher Focus Levels Compared to Silence

**FIGURE 6:**
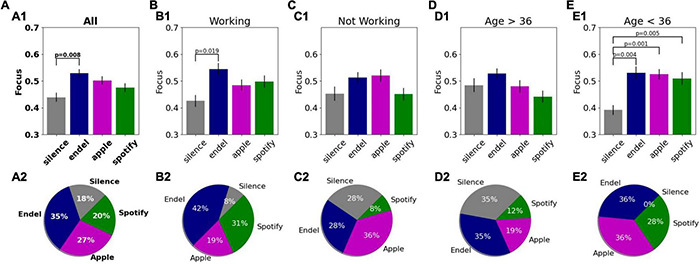
Comparison of the brain decoded focus during the Preferred Task while listening to different sounds. (Top row) Average focus levels for each sound stream during the Preferred Task for each group of interest, including statistical results. Error bars are standard errors. (Bottom row) Distribution of the best session (highest focus on average) for each participant per group. The groups of interest are: **(A)** all participants (51), **(B)** participants who were working during the Preferred Task (26), **(C)** participants who were not working (25-reading, knitting, playing, etc.). **(D)** Participants above 36 (26). **(E)** Participants below 36 (25).

Using the validated focus models which output five measurements per second (5 Hz), we then compared between the average focus levels elicited by the audio listed to during the Preferred Task. The background audio condition was found to have a significant effect (top row in Table 1, F(3,150) = 4.28, *p* = 0.006, statistical methods for details) on the elicited focus level and the *post hoc* tests [Holm–Bonferroni correction] revealed that streaming soundscapes (Endel app) were significantly higher compared to silence [Figure 6A1 and Supplementary Table 1; *M* = 0.090, SE = 0.027, *t*(50) = −3.38, *p* = 0.008], while streaming music using Apple or Spotify did not have an effect [Apple: *t*(50) = −2.37, *p* = 0.11, Spotify: *t*(50) = −1.24, *p* = 0.65].

**TABLE 1:** Results of a one-way repeated measures ANOVA performed on each subgroup, comparing the average brain decoded focus levels of each sound stream during the Preferred Task.

Group	N	F	p
All	51	4.28 (3,150)	0.006
Working	26	3.74 (3,75)	0.014
Not working	25	1.91 (3,72)	0.14
Age >36	26	1.81 (3,75)	0.15
Age <36	25	6.97 (3,72)	<0.001

*Sound most significantly affected those below 36 years old.*

For 35.3% of participants the soundscape session produced their highest focus level, while for 27.5% of participants the Apple playlist produced their personal highest focus level. For 19.6% of participants Spotify was best for producing focus and for 17.6% silence was (Figure 6A2, the details sorted focus levels per participant are shown in Supplementary Figure 4). To gain a better understanding of the conditions where audio affected focus, we next split the participants into subgroups of interest and repeated the statistical analysis. We first asked whether the focus level difference is task dependent. During the Preferred Task, 51% of the participants (26) chose to work, while the remainder (49%) read a book (29.4%), played games (9.8%), or performed other various tasks (e.g., knitting, 9.8%). To assess the effect of audio on focus levels during these different tasks, we split the participants to the ones who worked and those that did other tasks. We found that for the “working” group, the focus level elicited by Endel soundscapes was higher compared to silence [Figure 6B; *M* = 0.12, SE = 0.04, *t*(25) = 3.26, *p* = 0.017], while for the “not-working” group there was no difference [Figure 6C and Supplementary Table 1; *M* = 0.06, SE = 0.04, *t*(24) = 1.552, *p* = 0.447]. These results suggest that the focus level differences between Endel and Silence are task-dependent, where audio was particularly beneficial for specific types of tasks, namely, “working.”

We next split the participants into two age groups according to the median age (36 years). We found that for the younger participants (age < 36, *N* = 25), all audio types were superior to silence for producing elevated focus levels [Figure 6E and Supplementary Table 1; *M* = 0.14, 0.13, 0.12, SE = 0.04, 0.03, 0.03, *t*(24) = 3.79, 4.49, 3.67, *p* = 0.004, 0.001, 0.005 for Endel, Apple, and Spotify, respectively] while for the older participants (Figure 6D; age > 36, *N* = 26), there was no difference between audio and silence. The focus level differences were therefore found to also be age-dependent.

### Time Series Analysis of Focus Dynamics Reveal Differences Between Audio and Silence

**FIGURE 7:**
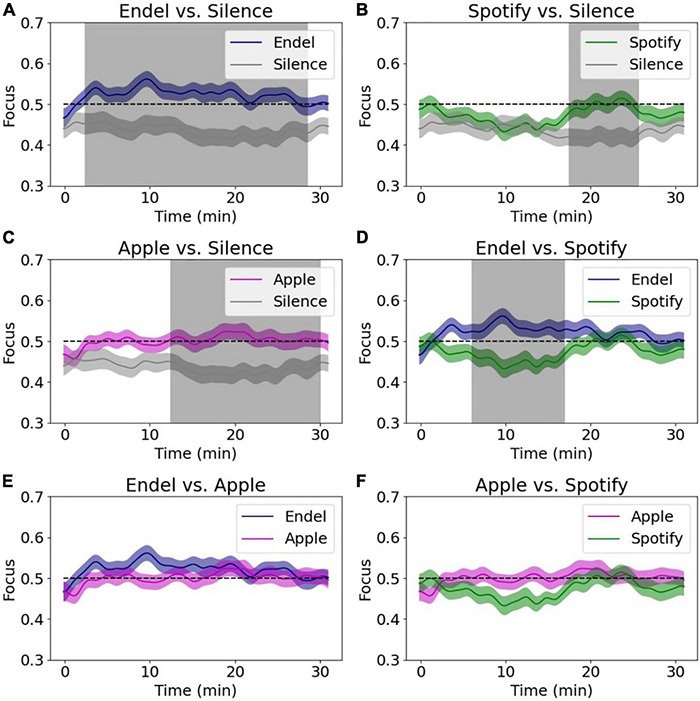
Comparing brain decoded focus dynamics during the 30 min of the Preferred Task. Each subfigure shows a comparison between two sound streams, while the gray areas are the timings with a significant difference (*p* < 0.05 corrected, see statistical methods for details).

Exploiting the high temporal resolution of the focus measurements, we compared the focus dynamics to each audio stream that played during the 30 min of the Preferred Task (Figure 7 and Table 2). When comparing Endel’s soundscapes vs. Silence (Figure 7A), we found that the focus level elicited by Endel’s soundscape was higher 87% of the time, a separation whose significance started after 2.5 min of listening. In addition, although on average there was not a significant difference, the focus level elicited by Apple’s playlist was higher than Silence 60% of the time, starting at 12.5 min (Figure 7C), and the focus level elicited by Spotify’s playlist was higher than Silence 27% of the time, starting at 17 min (Figure 7B). Focus elicited by Endel’s soundscape was higher than Spotify’s playlist in 37% of the time, starting at 6 min (Figure 7D).

**TABLE 2:** Summary of focus time dynamics comparison, showing for each pair the percentage of time and time segments with significant difference (where 100% = 30 min).

Pair	Significant difference (% session)	Significant segments (minutes)
Endel-Silence	87	2.5−28
Apple-Silence	60	12.5−30
Spotify-Silence	27	17.5−25.5
Endel-Apple	0	
Endel-Spotify	37	6−17
Spotify-Apple	0	

### Focus Levels Can Be Predicted by Properties of Audio

Seeing that background sound had an effect on focus levels, we go further and ask whether music and soundscapes can be composed according to a formula to increase focus levels. Meaning, can we understand which audio properties drive focus well enough to predict focus levels from exclusively an analysis of the properties of the sounds within the audio content?

Leveraging the high temporal resolution of the brain measurements, we generated a prediction model which predicts the brain-based focus level from features extracted from the audio signal alone. Raw audio files containing the Apple and Spotify sessions were used to extract different sound properties with a running sliding window of 30 s. The personalized soundscape session (Endel) was not used in this analysis because the real-time streaming did not allow saving the raw audio files that were consistent across participants. We combined multiple audio features to generate an audio data based model that predicts focus levels (see section “Materials and Methods”).

**FIGURE 8:**
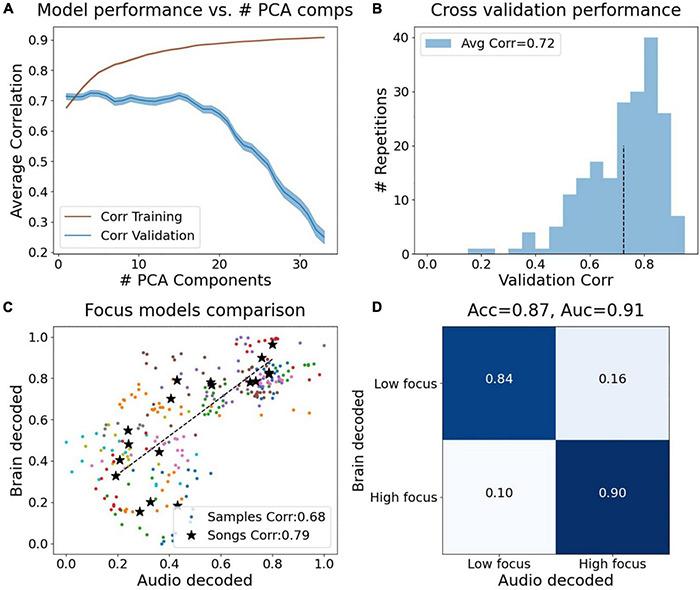
Results of predicting brain decoded focus from audio features. **(A)** Training and validation correlations vs. number of PCA components used as audio features, using backward elimination in each iteration the component with the smallest weight was eliminated. **(B)** Histogram of the validation correlations using four PCA components (PC1, PC2, PC9, and PC16). The average validation score is 0.72. **(C)** Brain decoded focus (*y*-axis) vs. audio decoded focus (*x*-axis) for all samples and average per song. The audio decoded scores were calculated across iterations they were part of the validation songs. **(D)** Confusion matrix after thresholding the focus predictions to classify between low and high focus. Classification accuracy obtained: 88% (area under ROC curve: 0.91).

Figure 8 shows the audio model performance in predicting the brain decoded focus levels. As explained in section “Materials and Methods,” Figure 8A shows the validation correlation and the training correlation for the backward elimination procedure, showing the best correlation on the validation set (≪*cps*:*it* > *corr* < /*cps*:*it*≫ = 0.72) is with four PCA components (PC1, PC2, PC9, and PC16). In addition, using only a single component (PC1) yielded a very close result (≪*cps*:*it* > *Corr* < /*cps*:*it*≫ = 0.71). The distribution of these correlations can be seen in Figure 8B. Comparing the brain decoded focus scores to the average predicted scores of each sample (across models the sample was part of the validation set), yielded a correlation coefficient of Corr(274) = 0.68, *p* < 1e-5, and Corr(16) = 0.79, *p* < 1e-4 when averaging the samples within each song (black points, Figure 8C). Using the songs scores, one can apply these prediction models to assemble more successful playlists for enhancing focus based on existing songs. Figure 8D shows that if we threshold the sample scores to output a binary prediction (low/high focus), the audio model reaches 87% accuracy in predicting the brain based focus (area under ROC curve = 0.91).

**FIGURE 9:**
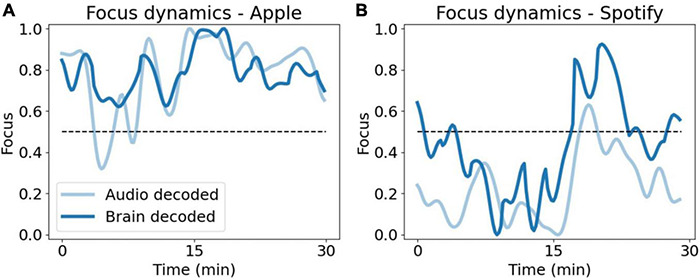
Comparison of focus model dynamics. Smoothed dynamics of brain decoded focus (dark blue) and audio decoded focus (light blue), during 30 min of the Preferred Task for Apple **(A)** and Spotify **(B)**. The audio decoded dynamics here was obtained using a model trained on all data (all songs).

For visualization of the decoded dynamics during the 30 min of the Preferred Task, we next trained the audio model using all 18 songs (without cross validation) and the four PCA components as features and projected it on the Apple (Figure 9A) and Spotify (Figure 9B) sessions.

### Analysis of Audio Properties Can Be Used to Understand Song Performance

To gain additional insights about the effects that different audio types have on human focus, we used the trained audio model to infer focus values for songs and sounds which were not played during the brain recording experiment. Meaning, we obtained a focus score and dynamics for chosen songs based solely on the properties of the sounds they contained. Here we selected audio examples that challenged the validity of the audio model based on their categorical exclusion from the brain recording experiment. A future approach can include these different genres as controls for further brain measurement validation studies. For example, soundscapes which are not personalized (taken from the playlist: “Focus: Calm Clear Morning”), natural sounds which are commonly used for increasing focus (such as white noise, waves, rain, taken from: https://mc2method.org/white-noise/), and popular songs from other music genres (classical music, electronic, pop, rock, jazz, and hip-hop) were used.

**FIGURE 10:**
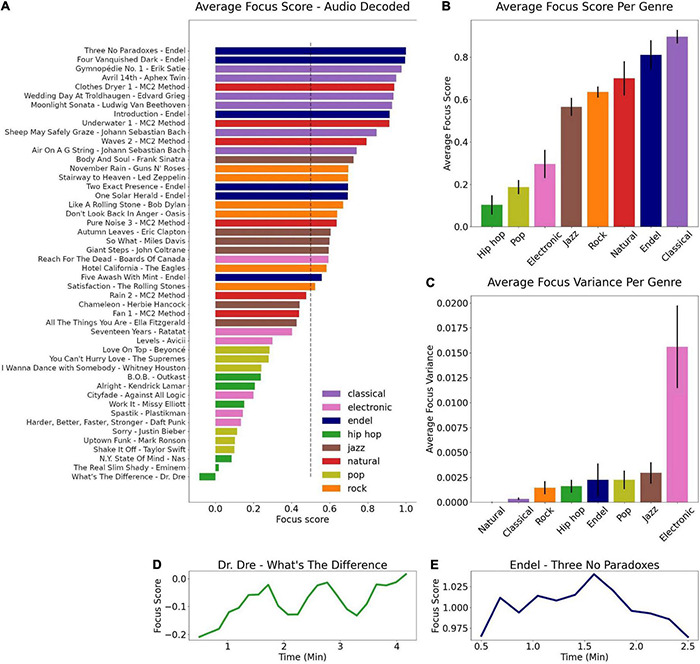
Projecting new songs into the trained audio model. **(A)** Sorted focus scores per song obtained by the audio model, colored by genre. **(B)** Average focus score per genre, sorted from the genre with the lowest score (hip-hop) to the highest (classical). **(C)** Average focus variance per genre, sorted from the genre with the lowest variance (natural) to highest (electronic). Focus dynamics for the song with the lowest focus score **(D)** and the highest **(E)**.

Figure 10A shows the predicted focus score based on the audio model which took into account only the properties of the audio itself. Songs are sorted from the highest focus evoking song (Endel – Three No Paradoxes) to the lowest (Dr. Dre – What’s The Difference). The top two songs are Endel soundscapes which are not personalized, a finding which strengthens our main result since it implies that the high focus scores elicited by Endel’s soundscape were not solely a byproduct of personalization but also related to the core audio content the personalized compositions were created from. Figure 10B shows the sorted focus scores averaged across genres, where notably sounds from classical music and natural sounds contained properties that predicted the highest focus levels. In contrast, pop and hip-hop songs predicted relatively low focus scores. Although we do not have ground truth focus labels for these songs based on real human brain data, given the relatively high scores of the audio which were known to have generated increased focus objectively in the experimental data, we can conclude that there is a consistent validity to the model. Future research can gather ground truth labels for these songs and evaluate the model mathematically in this context.

Analyzing the average within-song variance across different genres revealed that the model predicts the largest variance on average for electronic sounds (Figure 10C), while the lowest variance was found for natural sounds. The variance can be interpreted as a range of focus dynamics, where the focus dynamics of the electronic sounds are observed to change dramatically during a given song (Supplementary Figure 5), confirming the preference for a tool which outputs dynamics with a high temporal resolution when studying such audio content and not the oversimplification of post-song surveys or other low resolution methods. Figures 10D,E show the focus dynamics for the song with the lowest focus evoking score and the highest. The dynamics for all songs can be seen in Supplementary Figure 5.

## Discussion

“The soundscape of the world is changing. Modern man is beginning to inhabit a world with an acoustical environment radically different from any he has hitherto known” said the composer R. Murray Schafer, presaging the time we live in now when the sounds available to us continue to multiply by the day. As we have an increasing number of options to modulate our auditory lives by, a handful of take-aways from this study standout:

### Objective, Brain-Based Measurement of Focus Is Possible in Everyday Environments

Although the effects of audio on the human brain can be subtle in measured brain signals when judging by the changes produced in raw electromagnetic currents, they are robust and highly quantifiable with effectively trained algorithms, as shown here. Classifying emotional and attentional responses is particularly useful when done at the sub-second temporal resolution since it allows one to track dynamics continuously over time at the same timescale as the brain functions that impact perception and behavior. Furthermore, sub-second resolution into reactions that occur within ecologically valid conditions, like a participant’s home, model the real world in an everyday manner that is missing from experiments that take place within laboratories or other controlled test locations.

In this study we demonstrated that brain decoding algorithms processing data from a non-invasive, consumer brain-computer interface device, are able to deliver sub-second temporal resolution with a high degree of accuracy (approximately 80% match to self-report, Figure 5C) at people’s own homes. Since there are inherent biases in subjective self-reporting for experience (Kahneman et al., 1999; Mauss and Robinson, 2009), when mapping physiological signals to self-reported experiences, as done here, there is an upper boundary for accuracy beyond which any model must be deemed to over fit self-reported values and incorrectly represent the information observed in physiological signals. According to a recent review (Larradet et al., 2020) which summarizes multiple peer-reviewed studies that predict self-reported emotions from physiological signals, the average accuracy reported was ∼82%. Given this average and the experimental conditions here – a small number of sensors, at home recordings, simple self-report scales – the achieved accuracy was satisfactory for drawing deeper conclusions on properties of audio since it aligns with state-of-the-art emotion recognition accuracies in the context of audio as a stimulus used elsewhere in controlled laboratory environments (Tripathi et al., 2017, 81.41 and 73.35% for two classes of Valence and Arousal, respectively).

A key benefit of the current approach is that this method of high temporal resolution brain measurement can be performed reliably outside of traditional laboratories. In this current study not a single laboratory or facility was used for data acquisition. Instead, 18–65 years olds across the United States received a kit in the mail that included a head-wearable device, and they experienced music playlists and personalized soundscapes while they recorded their own brain signals in the comfort of their own home at the times of their choosing. In other words, all participants were in their natural habitat, wearing headphones and a headband that did not interfere with their experience, and they went through the study at their own pace, factors which altogether lend the research a rare degree of ecological validity.

### Focus Is Increased Most by Personalized Soundscapes

Within the at-home environment of this study, personalized, engineered soundscapes were found to be the best at increasing participant focus levels (Figure 6A). After 2.5 min, on average, listeners of the personalized soundscapes experienced a meaningful increase in their focus level, while for music playlists it took approximately 15 min to gain a similarly appreciable increase (Figure 7). The audio effect on focus levels was found to be task dependent, where soundscapes increased focus levels most in participants who were working (Figure 6B). For participants who were not working, no significant difference was found. This result suggests that willful orientation of attention toward work tasks may have created a brain context especially suited to modification by audio. While engaged in work, participants may also have been more prone to distraction and thus more impacted by the positive uplift of audio compared to when engrossed in reading or playing a game which may have contained more intrinsic motivation to stay focused on.

One limitation of this current study is that it did not allow us to disentangle the effects of personalization of sounds on the listener, since pre-recorded soundscapes were not tested. Equivalently, a comparison of personalized soundscapes to personalized music playlists, where audiences either made their own playlist for focus or were allowed to skip songs whenever they wanted, will likely contribute to a more complete understanding of how audio properties correlate with emotion and attention changes. Follow-up research will incorporate these variables. An additional limitation was the inability to reach conclusions regarding gender-dependent effects which was at least partially due to this study’s slightly imbalanced data set. Despite efforts to recruit a balanced group of participants, which included even outreach to all genders, enrollment was done on a rolling basis as necessitated by the data collection timeline for the research and ultimately the female subgroup was statistically underpowered in the analysis.

In future research, especially for closed loop, real-time testing, balanced participant sets will be important for reaching more detailed conclusions. Future research should also address whether any effects were introduced by the current study design’s sequence of tasks, since here we did not randomize the order of Preferred Task and validation tasks. The Preferred Task was always first and validation tasks after it intentionally, in order to allow for randomization of the background sound stimuli during the Preferred Task session which was done across four groups in this study. In future research it will be helpful to randomize the task order also to compare how different audio affects focus levels on different tasks according to a given task’s place within a sequence of tasks.

### Audio Preferences and Focus Effects Vary Between People

It is important to emphasize that the results reported here are audio effects on the average focus levels across a United States based population, and that there was a large variance in this effect between participants. Evidence for this large variance can be seen in Supplementary Figure 4 and in the age dependency effect (Figures 6D,E), where for the younger audience, all sounds increased focus while for the older audience, the sounds did not have any effect. These results are consistent with other studies showing personal preferences are critical for the improvements possible by audio (Cassidy and Macdonald, 2009; Huang and Shih, 2011; Mori et al., 2014). Due to this variety observed together with the highest focus being elicited by the personalized soundscapes, a next step will include closed-loop selections of sounds, where iterative sound testing is used per person to identify the significant parameters for maximizing focus for that person.

Personalized soundscapes specifically, and personalized audio in general, should be investigated further for their capacity to increase productivity, creativity and well-being as these attributes of human experience are associated with one’s ability to focus. For clinical populations as well, for example children with ADHD, the tailoring of sounds for this purpose of increased focus can be particularly impactful. It is possible that the seamlessness of the personalized soundscapes tested here, which played continuously without gaps in the sound like the music playlists had between songs, was also critical part of the observed effect on focus. At every juncture of the experience there is more to be learned, but at a high level, a main finding of this study is that there is a strong need for personalization of audio in order to most effectively achieve functional goals like increasing focus.

### Brain Decoded Focus Data Enabled a New Predictive Model Based on Audio Data Alone

Leveraging the high temporal resolution of the brain decoded dynamics, a focus prediction model based on the physical properties of audio was successfully trained, resulting in an accuracy score of 88% in predicting the brain decoded focus score from an audio decomposition that assessed 136 different properties of sounds as unique features (Figure 8). This model enabled a further examination of how sounds and different genres effects focus and allowed testing additional conditions, such as pre-recorded soundscapes and commonly used background sounds (e.g., white noise), as well as other genres (pop, rock, jazz, etc.). We found that the model predicted the highest focus scores for classical music, followed by engineered soundscapes and natural sounds. These results complement previous studies which showed natural sounds and classical music to be beneficial for learning and concentration (Davies, 2000; DeLoach et al., 2015; Angwin et al., 2017; Liu et al., 2021).

In contrast, the models predicted that genres such as pop and hip-hop produce lower focus levels (Figures 10A,B). It is possible that these sounds contain more distractors that attract attention away from other objects of attention, or that they contain types of sounds that the brain requires more resources to process (depending on familiar patterns, surprises, and more), leading to less resources available to perform other tasks. Sounds in these genres may also activate the reward system differently (Salimpoor et al., 2015; Gold et al., 2019), which can increase motivation to listen intently to the songs themselves rather than orient toward other tasks. Understanding the brain mechanisms that underlay the modified focus from these genres is beyond the scope of this current research, but the mapping found here can provide fruitful avenues for future brain imaging experiments that may be equipped to answer these questions.

The analysis here demonstrates a process in which we utilize the temporal resolution of brain-computer interface technology to generate a product where the neurotechnology is eventually out of the loop, resulting in a stand alone audio model which takes as an input a raw audio file and outputs a predicted focus score. This model can be used independently to generate focus playlists or to compose optimal soundscapes, and can further be improved by expanding to populations outside the United States and different age groups. In this way, the current research hearkens back to Pythagoras, who first identified the mathematical connection between a string’s length and it is pitch and believed the whole cosmos was a form of musical composition (James, 1995). We too see the rich mathematical models obtained in this study, by mapping audio properties to human experience, as a glimpse into the natural laws governing how we feel and think. The better these laws can be understood, the more empowered individuals will be to modulate their environments to suit their goals and states of mind. There remains much to figure out: while we as a species continue to cause a “shift in the sensorium,” we simultaneously experience that shift all over daily life and it is not clear where we as a species are headed. This study showed that audio has a distinct effect on our focus levels, and paves the way for designing sounds to help us focus better in the future.

## Conclusion

We studied the effects of audio on human focus levels using noninvasive brain decoding technology to gain a better understanding of the optimal audio properties for increasing focus levels in listeners. We combined a custom app (“Neuos Central”), portable fabric EEG-measuring headbands, and brain decoding technology (Neuos SDK) to enable us to obtain high temporal resolution focus dynamics from participants at home. Using the brain decoded focus dynamics, we then analyzed how various properties of audio impacted focus levels in different tasks.

We found that while performing a self-paced task for a long period of time (such as working), personalized soundscapes increased focus the most relative to silence. Curated playlists of pre-recorded songs by Apple and Spotify also increased focus during specific time intervals, especially for the youngest audience demographic. Large variance in response profiles across participants, together with task and age dependent effects, suggest that personalizing audio content in real-time may be the best strategy for producing focus in a given listener.

Finally, we generated an audio property based focus model which successfully predicts brain decoded focus scores from audio file alone as an input. Using this model, we extracted predicted focus scores from new songs based on audio decomposition and performed a genre analysis to develop new intuitions about the experimental findings and the sources of focus-producing audio content. We found that based on our model, engineered soundscapes and classical music are the best for increasing focus, while pop and hip-hop music are the worst.

The approach taken here can be adapted to include other emotions (e.g., enjoyment, anxiety, happiness, etc.), attentional parameters (“Flow state,” memory formation, etc.) and can be used to assess additional content as well (e.g., visual, ambient, olfactory, etc.), including interactive gaming and e-learning experiences where personalization and high temporal resolution measures of brain responses may be especially beneficial.

## Data Availability Statement

The dataset for this study is available through an open Git repository (https://bit.ly/3HbZd8n). Data includes the brain decoded focus dynamics for each participant together with scripts that run the statistical tests.

## Ethics Statement

Ethical review and approval was not required for the study on human participants in accordance with the local legislation and institutional requirements. The patients/participants provided their written informed consent to participate in this study.

## Author Contributions

AH, RK, NB-E, EK, and DF designed the experiment. AH analyzed the data. RK, NB-E, and DF advised on data analysis and statistics. SK, EK, and DF developed the app and software platform for data collection. AH and DF wrote the manuscript. RK and EK revised the manuscript. All authors approved the work for publication.

## Conflict of Interest

The authors were employed by the company Arctop Inc. This study received funding from Arctop Inc., and Endel Sound GmbH. The funders had the following involvement with the study: Arctop Inc., was involved in the study design, collection, analysis, interpretation of data, the writing of this article, and the decision to submit it for publication. Endel Sound GmbH was involved in the study design and provided audio stimuli used in the experiment.

## Publisher’s Note

All claims expressed in this article are solely those of the authors and do not necessarily represent those of their affiliated organizations, or those of the publisher, the editors and the reviewers. Any product that may be evaluated in this article, or claim that may be made by its manufacturer, is not guaranteed or endorsed by the publisher.
