# Supplementary material for: Measuring and Modeling the Effect of Audio on Human Focus in Everyday Environments Using Brain-Computer Interface Technology
Source: Front Comput Neurosci. 2022 Jan 27;15:760561. doi: 10.3389/fncom.2021.760561 (PMC8829886; doi:10.3389/fncom.2021.760561)
Supplement: Supplementary file 1 [file Data_Sheet_1.PDF]

Supplementary Material

1 Supplementary Figures and Tables

1.1 Supplementary Figures

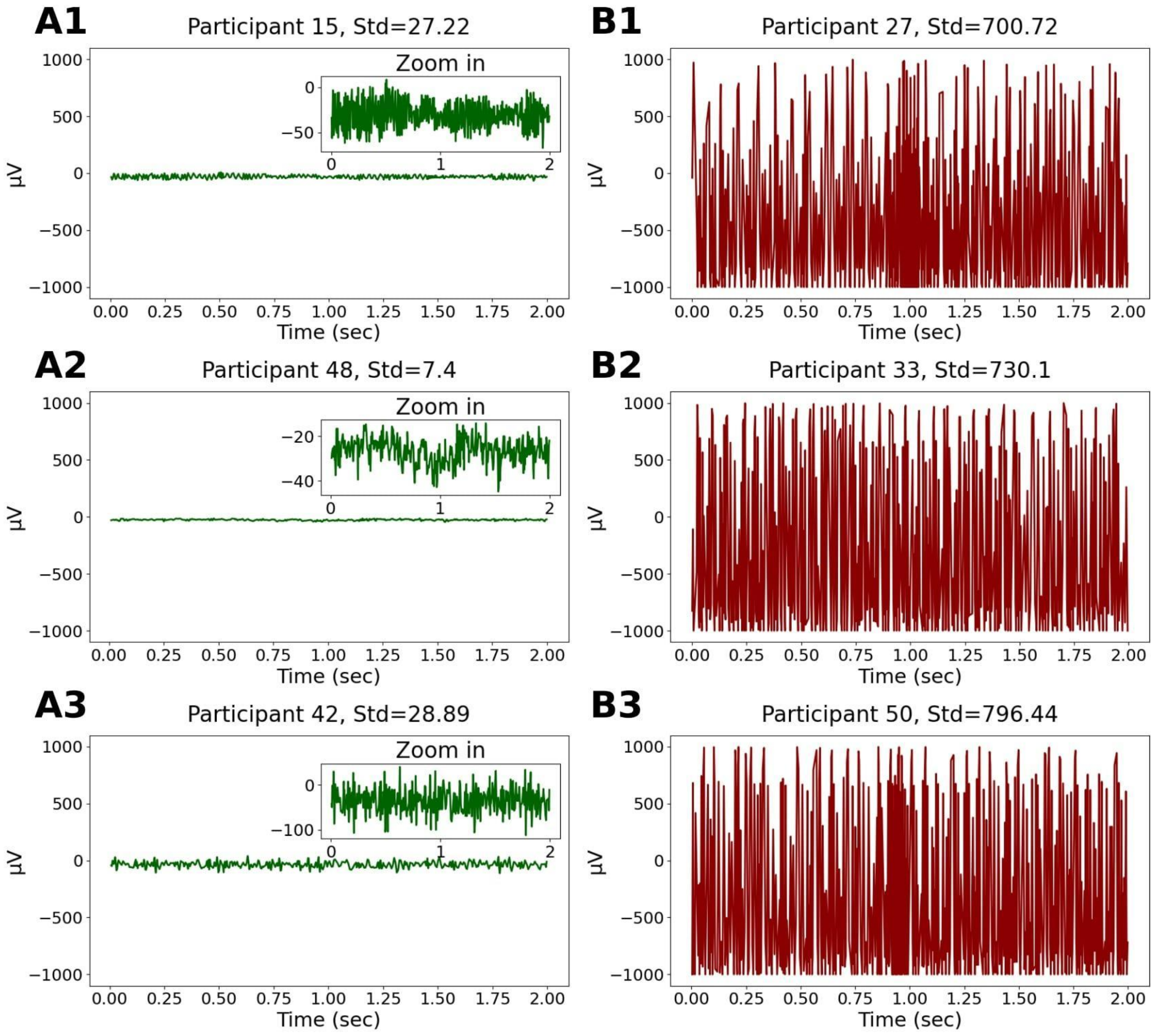

Supplementary Figure 1. Comparison of raw EEG data from excluded participants vs. valid participants. (A1-3) Examples of EEG raw segments (2 seconds) from valid participants (std<500). Inset figure shows the same segment zoomed in. **(B1-3)** Examples of EEG raw segments of problematic participants which misplaced the headband (std>500) and were excluded from the analysis.

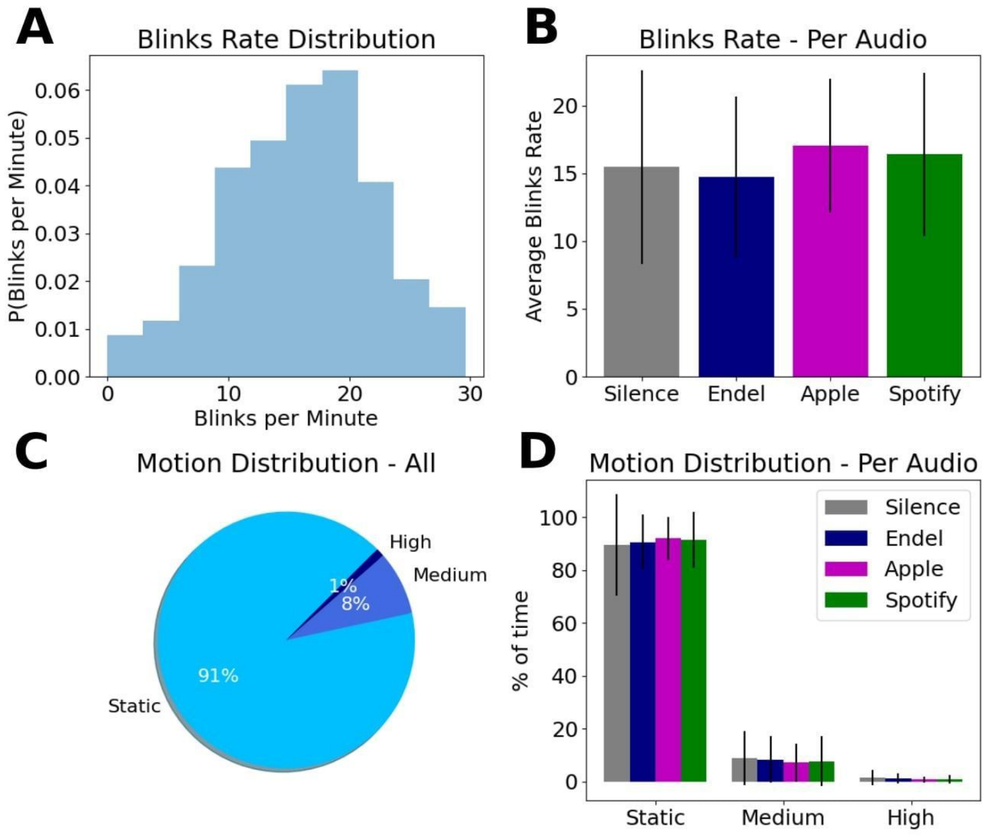

Supplementary Figure 2. Summary of motion and eye blinks effect. (A) Histogram of blinks rate (blinks per minute) from all participants. Average is 16 ±6 blinks per minute (a blink every 3.8 seconds). **(B)** Average blinks rate per audio type, showing similar rates **(C)** Motion distribution from all participants and sessions during the Preferred Task. 91% of the time participants were not moving significantly (static state). **(D)** Average percent in each motion state, divided by audio types, showing the same motion states statistics for all audio types.

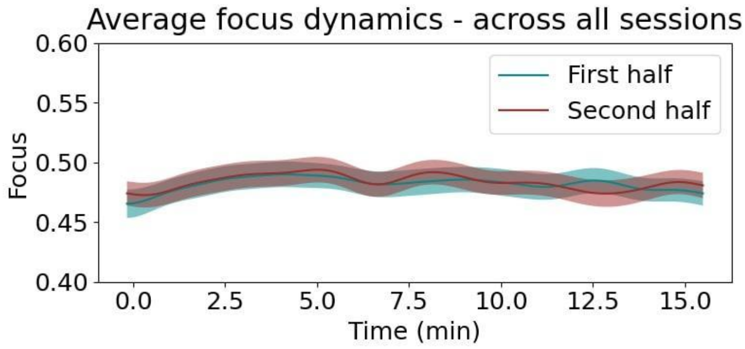

Supplementary Figure 3. Stationarity of focus dynamics. Average brain decoded focus dynamics across all participants and audio streams during the preferred task, splitted to the first 15 minutes (blue) and last 15 minutes (brown).

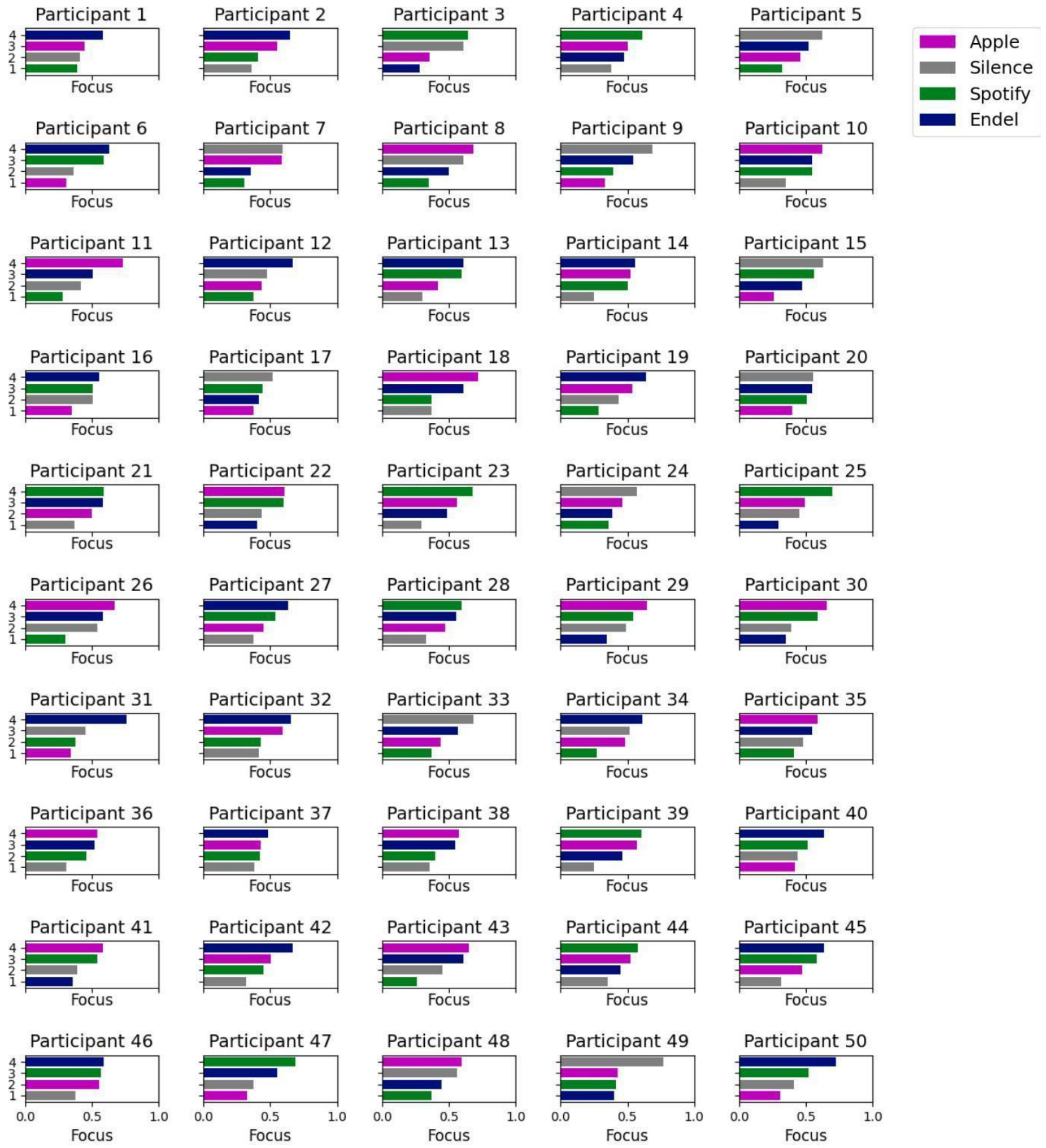

Supplementary Figure 4. Average Brain decoded focus levels for all participants in all sessions' Preferred Task. For each participant, the sessions are sorted from their highest average focus level (4) to the lowest (1), with colors representing the different experimental audio streams. The distribution of highest sessions is presented in Fig. 6B.

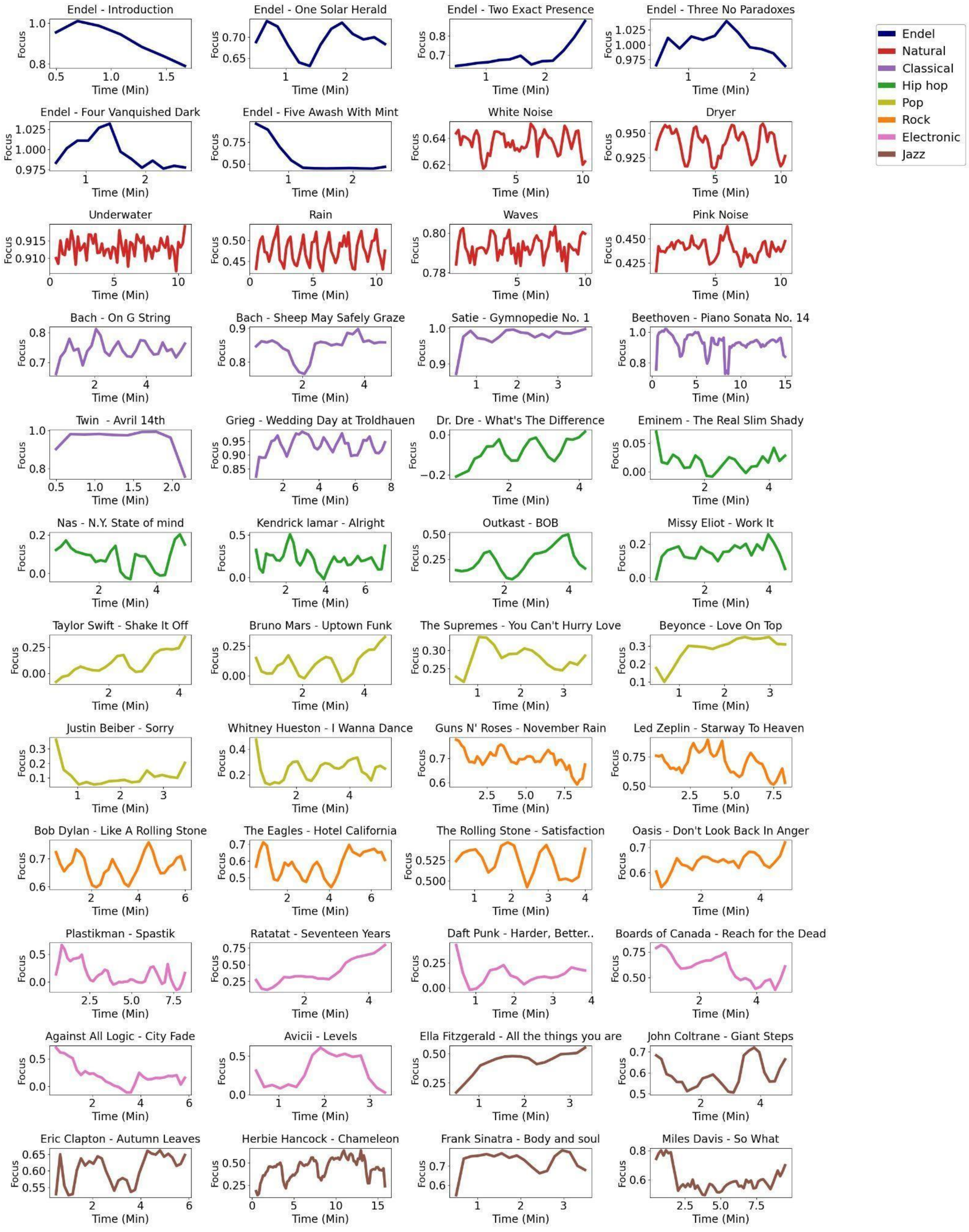

Supplementary Figure 5. Audio decoded focus dynamics for all songs. Each color represents a different genre according to the legend on the right. The properties of sound in each audio file were used exclusively to simulate listener focus dynamics - no brain measurements from real audiences were required.

1.2 Supplementary Tables

Supplementary Table 1. Results of post-hoc statistical tests, comparing all pairs of audio streams' focus levels for all and for each subgroup. P values are corrected using the Holm-Bonferroni method. Significant differences are colored in grey (p(holm)<0.05) and as long as the ANOVA test (Table 1) revealed significance differences.

Group	Pair	Avg	Ste	t	df	p (holm)
**All**	**Silence-Endel**	**-0.090**	**0.027**	**-3.38**	**50**	**0.008**
	Silence-Apple	-0.063	0.026	-2.37	50	0.107
	Silence-Spotify	-0.036	0.028	-1.24	50	0.653
	Endel-Apple	0.027	0.024	1.13	50	0.653
	Endel-Spotify	0.054	0.025	2.13	50	0.153
	Spotify-Apple	-0.027	0.026	1.03	50	0.653
**Working**	**Silence-Endel**	**-0.119**	**0.036**	**-3.26**	**25**	**0.017**
	Silence-Apple	-0.058	0.034	-1.68	**25**	0.317
	Silence-Spotify	-0.072	0.036	-1.172	**25**	0.292
	Endel-Apple	0.061	0.033	1.155	**25**	0.317
	Endel-Spotify	0.047	0.039	2.131	**25**	0.487
	Spotify-Apple	-0.014	0.034	1.015	**25**	0.687
**Not Working**	**Silence-Endel**	**-0.060**	**0.038**	**-1.552**	**24**	**0.447**
	Silence-Apple	-0.067	0.041	-1.650	**24**	0.447
	Silence-Spotify	0.002	0.044	0.045	**24**	1.000
	Endel-Apple	-0.007	0.034	-0.202	**24**	1.000
	Endel-Spotify	0.062	0.033	1.870	**24**	0.442
	Spotify-Apple	0.069	0.037	1.838	**24**	0.442
**Age > 36**	**Silence-Endel**	**-0.044**	**0.037**	**-1.17**	**25**	**1.000**
	Silence-Apple	0.005	0.039	0.12	**25**	1.000
	Silence-Spotify	0.043	0.042	1.01	**25**	1.000
	Endel-Apple	0.048	0.034	1.42	**25**	0.840
	Endel-Spotify	0.086	0.029	2.91	**25**	0.044
	Spotify-Apple	0.037	0.039	0.97	**25**	1.000
**Age < 36**	**Silence-Endel**	**-0.139**	**0.036**	**-3.79**	**24**	0.004
	Silence-Apple	-0.133	0.029	-4.49	**24**	0.001
	Silence-Spotify	-0.117	0.032	-3.67	**24**	0.005
	Endel-Apple	0.006	0.034	0.16	**24**	1.000
	Endel-Spotify	0.021	0.042	0.51	**24**	1.000
	Spotify-Apple	-0.015	0.035	0.44	**24**	1.000
